# Canal Configuration of Mesiobuccal Roots in Permanent Maxillary First Molars in Iranian Population: A Systematic Review

**Published:** 2016-11

**Authors:** Mandana Naseri, Mohammad Javad Kharazifard, Sepanta Hosseinpour

**Affiliations:** 1 Associate Professor, Department of Endodontics, School of Dentistry, Shahid Beheshti University of Medical Sciences, Tehran, Iran; 2 Epidemiologist, Dental Research Center, Dentistry Research Institute, Tehran University of Medical Sciences, Tehran, Iran; 3 Dental and MPH Student, Students’ Research Committee, Shahid Beheshti University of Medical Sciences, Tehran, Iran

**Keywords:** Anatomy, Dental Pulp Cavity, Molar, Maxilla, Review Literature as Topic

## Abstract

**Objectives:**

It is essential for clinicians to have adequate knowledge about root canal configurations; although its morphology varies largely in different ethnicities and even in different individuals with the same ethnic background. The current study aims to review the root canal configurations of mesiobuccal roots of maxillary first molars in an Iranian population based on different epidemiological studies.

**Materials and Methods:**

A comprehensive search was conducted to retrieve articles related to root canal configuration and prevalence of each type of root canal based on Vertucci’s classification for the mesiobuccal root of maxillary first molars. An electronic search was conducted in Medline, Scopus and Google Scholar from January 1984 to September 2015. The articles were evaluated and methods, population, number of teeth and percentage of each root canal type evaluated in each study were summarized in the data table. Websites such as http://www.magiran.com/ , http://health.barakatkns.com/journal-internal-list and www.sid.ir were used to search all related studies published in Persian.

**Results:**

Totally, out of nine studies conducted on the Iranian populations in nine provinces of Iran and 798 teeth, the Vertucci’s type I was the most common type (35.70%), followed by type II (30.37%), type IV (16.66%), type III (7.93%) and type V (2.61%).

**Conclusions:**

From this review article, it is concluded that the root canal morphology of mesiobuccal roots of maxillary first molars in the Iranian population predominantly has more than one canal. Therefore, careful evaluation of radiographs and anatomy of the pulp chamber is essential in order to achieve a successful root canal therapy.

## INTRODUCTION

Successful nonsurgical endodontic therapy is closely associated with locating all root canals, properly cleaning and shaping them both mechanically and chemically, and perfect obturation using appropriate sealant and root canal filling materials [1–5]. Therefore, it is essential for clinicians to have adequate knowledge about root canal configurations; although its morphology varies largely in different ethnicities and even in different individuals with the same ethnic background [6–8]. Root canal configuration is usually complicated and diverse [1,9, 10]. Based on the literature, in addition to ethnicity, age [11–14] and gender [15–17] can also influence these diversities.

Previous studies classified root canal morphology in various ways [12,18, 19]. First, in 1902, GV Black discussed human tooth morphology [20]. In 1969, Weine et al, [19] described a four-type classification method based on the pattern of division of the main root canal. In 1984, the details of human root canals were studied by Vertucci [18]. Vertucci [18] introduced a standardized and categorized method for differentiating the root canal variations into eight descriptive types [18]. This classification has been widely used in many studies [3 ,6,7,21 –25]. Fourteen new canal morphology types were added to these previous classifications [17]. However, many case reports indicate several variations that emphasize on complete evaluation of each case [26–30]. These variations make it difficult to accurately locate, clean and fill a root canal, and can lead to post-treatment complications and compromise the outcome of root canal therapy [31–33].

Although the most common root canal configuration for permanent maxillary first molars is three roots and four canals [34], several studies have reported uncommon anatomical variations in these teeth [27,35, 36]. Due to the wide buccolingual dimension of mesiobuccal root and presence of concavities on its mesial and distal surfaces, two canals are more common in these roots as compared to the distobuccal and palatal roots [1]. These facts have been confirmed by several studies on mesiobuccal roots of maxillary first molars (Table 1).

**Table 1:** Studies included in this systematic review and their reported prevalence of mesiobuccal canal configurations in maxillary first molars

** Author **	** Study Method **	** Race **	** Sample size **	^*^ Type I(%)	** Type II(%) **	** Type III(%) **	** Type IV(%) **	** Type V(%) **	** Type VI(%) **	** Type VII(%) **	** Type VIII(%) **
Alrahabi and Zafar [ 74 ] (2015)	Cone beam computed tomography	Not mentioned	100	29.4	47	11.8	11.8	0	0	0	0
Guo et al [ 37 ] (2014)	Cone beam computed tomography	African	628	28.3	26.3	1.1	41.9	2.4	NM	NM	NM
		American									
		Asian Hispanic									
		non-Hispanic white									
Yamada et al [ 38 ] (2011)	Micro-computed tomography	Japanese	90	44.4	22.3	24.4	8.9	0	NM	NM	NM
Zhang et al [ 40 ] (2011)	Cone beam computed tomography	Chinese	299	48	7.28	0	35.88	8.32	3	0	0
Neelakantan et al [ 41 ] (2010)	Cone beam computed tomography	Indian	220	51.8	5.5	0	38.6	0	0	0	0
Verma and Love [ 79 ] (2010)	Micro-computed tomography	Not mentioned	20	10	15	0	15	10	15	5	0
Pattanshetti et al [ 42 ] (2008)	Clinically and radiographically	Kuwaiti and non-Kuwaiti	110	57.7	33.6	8.7	0	0	0	0	0
Rwenyonyi et al [ 43 ] (2007)	Injection of Indian ink	Ugandan	221	75.1	4.1	0.9	11.3	5.8	1.4	0.9	0
Smadi and Khraisat [ 46 ] (2007)	Injection of Indian ink	Jordanian	100	22.6	27.8	2	35	1	7.3	3	0
Alavi et al[ 44 ]2002	Injection of Indian ink	Thai	268	32.7	17.3	1.9	44.2	1.9	7.7	3.1	1.5
Ng et al.[ 45 ] (2001)	Injection of Indian ink	Burmese	239	30	25.5	1.1	33.33	6.7	NM	NM	NM
Weine et al [ 39 ] (1999)	Files in place of extracted teeth	Japanese	300	42	24.2	30.4	3.4	0	0	0	0
Fogol et al [ 15 ] (1994)	Clinically and radiographically	Not mentioned	208	28.9	39.4	31.7	0	0	NM	NM	NM
Kulid and Peters [ 80 ] (1990)	Files in place of extracted teeth	Not mentioned	51	4.8	49.4	45.8	0	0	NM	NM	NM

* Evaluations were performed according to the Vertucci’s classification

In 2014, Guo et al, [37] reported types I and II patterns in 28.3% and 26.3% of these roots, respectively in African Americans, Asian Hispanics and non-Hispanic Whites. In two different studies on the Japanese, type I (44.4% and 42%) was the most frequent type, followed by type II (24.2% and 22.3%) [38,39]. Moreover, among the Chinese [40], Indian [41], Kuwaiti [42], Ugandan [43], Thai [44] and Burmese [45] populations, the most frequent type of canal in the mesiobuccal root of the maxillary first molars was type I followed by type II. However, these frequencies vary widely among these populations, so that in the Ugandan population, the prevalence of type I was 75.1% [43], while in the Burmese population, it was 30% [45]. On the other hand, Smadi and Khraisat [46] reported type II as the most frequent (27.8%) type among the Jordanian population.

In addition, a wide variety of methods have been used to investigate root canal morphology. The laboratory techniques include clearing techniques using decalcification [47] and injection of Indian ink [44,48, 49], hematoxylin dye [18], Chinese ink [50], metal castings [52,51], in vitro radiography [12,13, 53], in vitro macroscopic examination [36], grinding or sectioning [19,54] and scanning electron microscope examination [55]. Moreover, computed tomography (CT), spiral CT, micro CT and cone beam CT (CBCT) have been used for clinical investigations [4,57, 56]. All these methodological and biological factors contribute to variations in the reported prevalence.

The current study aims to review the root canal configurations of mesiobuccal roots in maxillary first molars among the Iranian population based on different epidemiological studies.

## MATERIALS AND METHODS

### Literature search and data extraction:

A comprehensive search was conducted to retrieve published and unpublished articles related to root canal configuration and prevalence of each type of root canals based on the Vertucci’s classification [18] in mesiobuccal root of maxillary first molars. An electronic search was conducted in Medline, Scopus and Google Scholar from January 1984 to September 2015 without language limitation in publications with available full texts using the following keywords: root canal anatomy, root configuration, root canal morphology and maxillary first molars. Moreover, similar search strategy was also applied for the Cochrane database and manual searches, including journals and reference lists. Two independent reviewers retrieved articles according to the defined keywords. They also performed initial screening on titles and abstracts of the selected articles according to the pre-defined eligibility criteria. Disagreement between reviewers was resolved by discussion and if still remained, a third person was consulted.

### Eligibility criteria:

All in vitro studies evaluating the canal configuration of mesiobuccal roots of permanent maxillary first molars were included in this study. Clinical studies and those assessing other teeth were excluded. Studies only conducted on the Iranian population, which used the Vertucci’s classification were included and other populations and methods of classification were excluded.

A total of 573 studies were found in the preliminary search. Then, their titles and abstracts were assessed to determine appropriate and related articles. After exclusion of irrelevant studies, 78 articles remained. Then, the full texts of the selected articles were obtained and reviewed. From each study, the methodology, sample size, sampling location and prevalence of different types of root canal configurations were extracted. Among the studies, 16 articles remained with their data and classification based on the Vertucci’s classification and only two studies had been conducted on the Iranian population [22,25].

Websites such as http://www.magiran.com/ , http://health.barakatkns.com/journal-internal-list and www.sid.ir were used to search all related studies published in Persian. From a total of 31 articles found as such, eight met our inclusion criteria. In addition, to collect unpublished or published regional data related to our study, a request was sent to all dental research centers and dental schools in Iran and nine studies were obtained as such. Finally, Data were collected on: 1) Author and year of publication; 2) Type of study; 3) Method of study; 4) Region of study; 5) Sample size, and 6) Type of root canal morphology.

## RESULTS

### Included studies:

Among 573 studies, in which anatomy and morphology of maxillary first molars and mesiobuccal canals were evaluated, 78 studies on root canal configurations were selected.

Anatomy and morphology of human extracted teeth were evaluated after access cavity preparation in included studies. Reference lists of included studies were evaluated to identify any potentially relevant article. Ten studies which were conducted on mesiobuccal canal configurations of maxillary first molars in the Iranian population met the inclusion criteria with a total of 649 teeth (Table 2). All of them were evaluated with clearing technique, direct observation, CBCT and radiography after cavity or section preparation.

**Table 2:** Studies included in this systematic review and their reported prevalence of mesiobuccal canal configurations in maxillary first molars in the Iranian population

** Author **	** Study method **	** City **	** Sample size **	^*^Type I(%)	** Type II(%) **	** Type III(%) **	** Type IV(%) **	** Type V(%) **	** Type VI(%) **	** Type VII(%) **	** Type VIII(%) **
Faramarzi et al [ 60 ]	Cone-beam computed tomography	Hamadan	156	30.77	49.36	0	19.87	0	NM	NM	NM
Rouhani et al [ 22 ]	Cone-beam computed tomography	Tehran, Mashhad, Tabriz, Bandar Abbas, Isfahan	125	46.4	14.4	9.6	3.2	3.2	17.6	5.6	0
Ezoddini et al [ 23 ]	Cone-beam computed tomography	Yazd	30	40	33.33	20	6.67	0	NM	NM	NM
Adel et al [ 24 ]	Observation	Qazvin	114	21.9	50.8	0.8	21.3	2.6	2.6	NM	NM
Shahi et al [ 25 ]	Injection of Indian ink	Tabriz	37	37.96	24.08	0	24.08	9.5	4.38	0	0
Ashofteh Yazdi et al [ 59 ]	Observation and radiography	Tehran	105	24.8	32.4	39	3.8	0	0	0	0
Sadeghi et al [ 58 ]	Staining and clearing	Kerman	50	20	32	4	40	4	NM	NM	NM
Naseri et al [ 62 ]	Cone-beam computed tomography	Tehran	149	10.1	32.9	10	35.6	11.4	0	0	0
Naseri et al [ 61 ]	Stereomicroscopy	Tehran	32	87.5	0	0	12.5	0	NM	NM	NM

Total			798	35.70	30.37	7.93	16.66	2.61			

* Evaluations were performed according to Vertucci’s classification

** NM: Not mentioned

### Data summary of included studies:

In 2004, Sadeghi and Sadr Lahijani [58] demonstrated that the most frequent type of root configuration among mesiobuccal roots of maxillary first molars was type IV (40%) in Kerman population. In their study, 50 human maxillary first molars were investigated by staining and clearing to determine the type and number of root canals and also the presence of accessory and lateral canals, which had a prevalence of 32% and 8%, respectively. One-hundred and five extracted maxillary first molars were evaluated in another study [59], in which after cavity preparation, the teeth were immersed in 1% fuchsin, incubated and root canal configurations were assessed by radiography and also observation of cross sections. The most common type was type III (39%). Shahi et al, [25] performed demineralization and Indian ink injection and evaluated the specimens by the use of a magnifying-glass at x5 magnification. They concluded that single root canal configuration, that is, type I, is more frequent in north-western Iran (37.96%).

In 2009, Adel and Hamzehnejad [24] conducted a study on Qazvin population, in which 114 extracted permanent maxillary first molars were investigated. All the specimens were sectioned at the cementoenamel junction. After injection of Indian ink into the root canal system, teeth were cleared, demineralized and dehydrated. Each specimen was evaluated and classified by two calibrated dentists. The results indicated type II as the most common type.

A total of 311 maxillary first molars were evaluated by CBCT in three separate studies in different parts of Iran (Hamadan [60], Tehran, Mashhad, Tabriz, Bandar Abbas, Isfahan [22] and Yazd [23]) in 2014–2015. The thickness of each section was different in these studies; in one study, it was 1mm [60], 2mm in another one [23] and not mentioned in the study by Rouhani et al, [22]. Among these studies, Ezoddini et al, [23] demonstrated the presence of second mesiobuccal canal in 60% of the specimens; it was in the coronal third of the teeth in 55.55% of the cases. Rouhani et al, [22] reported type one as the most common canal morphology for both first and second maxillary molars. Moreover, they mentioned that the prevalence of root fusion in maxillary first and second molars was 2.4% and 8.8%, respectively.

Faramarzi et al, [60] also reported the presence of second mesiobuccal canal in 69.23% of maxillary first molars. However, Naseri et al, [61] indicated the presence of second mesiobuccal canal in 12.5% of teeth, which may be due to small sample size (32 maxillary first molars). In total, as shown in the results of the present study, type I was the most common canal configuration of mesiobuccal root in maxillary first molars in the Iranian population (38.55%), followed by type II (29.54%), type IV (16.41%), type V (12.41%) and type III (6.92%).

## DISCUSSION

Achieving a successful root canal therapy requires adequate knowledge about root canal configurations. Diversity of root canal morphology and possible variations in mesiobuccal root of maxillary first molars can affect the outcome of treatments [63,64]. The present study reviewed published and unpublished epidemiological studies, which investigated mesiobuccal root configurations in several provinces of Iran. These results show the significance of negotiating for extra canals in mesiobuccal root of maxillary first molars, especially in the Iranian population with 61.45% probability of having more than one root canal. It has been largely reported that the most common type of permanent maxillary first molar root is three separate roots [65] and rare cases have been reported with more than three separate roots [66]. Many factors can contribute to the number of root canals.

In 1999, Weine et al, [39] conducted an investigation among a Japanese population to detect the incidence of second mesiobuccal canal; their results were similar to those of other studies. However, they mentioned the impact of age on the incidence of second mesiobuccal canal. According to their results, fewer canals were detected in the mesiobuccal root due to calcification in older people, and several other studies confirmed these findings [15,55, 67]. None of the studies included in our present review assessed the possible correlation between age and root canal anatomy and further studies are needed in this regard on the Iranian population. Although it seems that one mesiobuccal root is the most frequent type based on the external anatomy of these teeth, type I canal configuration is not the most common. In 2006, Cleghorn et al, [68] investigated the root anatomy of maxillary first molars in a comprehensive review article.

A total of 8,400 teeth and all palatal, distobuccal and mesiobuccal canals were analyzed in their study. It seems that the prevalence of two palatal canals is very low all over the world [17,69, 70]. Studies on mesiobuccal root morphology comprise a large part of dental literature. This is due to the significantly high prevalence of more than one root canal in a broad range of diversities [71].

In 2004, Sert and Bayirli [17] investigated the impact of gender on the prevalence of different types of canals among a Turkish population. They showed that single canal (type I) in mesiobuccal root was seen in 10% of females as compared to 3% of males. Iranian researchers did not mention any statistically significant association between gender and internal anatomy variations of teeth. However, several studies reported results in contrast to these findings and did not mention any correlation between gender and the type of root canals [15,17,67 ,72]. In two separate studies, different techniques of morphology evaluation (in vitro versus clinical) were compared [54,73]. In both studies, significantly higher prevalence of second mesiobuccal canal was reported when an in vitro technique was used. Such differences are partly attributed to the definition of a “treatable canal” used in clinical practice and studies [15,74] versus visible root canal morphology in clearing studies [17, 18,44, 48,72]. Furthermore, spiral CT or CBCT recently used in several studies seems to be the most reliable method for determination of both internal and external morphology of root canals [37,41,75–79].

In this review, most of the mesiobuccal roots in the Iranian population had more than one canal. These findings are in agreement with those of several studies conducted around the world [15 ,46,75,80,81]. The highest prevalence of one canal (type one; 75.1%) in the mesiobuccal root of maxillary first molars was reported by Rwenyonyi et al, [43]. A wide variety of two or more canals has been reported: 25% [26], 55% [2], 58% [9], 73.6% [17] and 78% [48] in different studies pointing to the possible impact of ethnicity. In the present review and three other studies [60,23, 25], type II was the most frequent type of root canals. However, in other studies [22,24,58,59,61], other anatomical types were more common. These differences might be due to various techniques and populations. Recently, the application of surgical operating microscopes or loupes increased the clinical determination of second mesiobuccal canals [71,74,81,82 ]. On the other hand, Davis et al, [83] demonstrated that intact canal walls were seen in standard instrumentation by injection of silicone impression. Moreover, missed or not fully instrumented canals can reduce the overall long-term prognosis of root canal therapy [84]. Finally, these findings indicate that the internal root canal morphology of maxillary first molars, especially the mesiobuccal roots, has never been straight forward.

## CONCLUSION

Our study demonstrated that mesiobuccal roots of maxillary first molars in the Iranian population predominantly have more than one canal. Other morphologies have been rarely reported. Therefore, careful evaluation of radiographs and the anatomy of the pulp chamber is essential to achieve a successful root canal therapy. Clinicians should focus on each case individually in addition to their anatomical knowledge.
